# Accurate magnification determination for cryoEM using gold

**DOI:** 10.1016/j.ultramic.2023.113883

**Published:** 2024-02

**Authors:** Joshua L. Dickerson, Erin Leahy, Mathew J. Peet, Katerina Naydenova, Christopher J. Russo

**Affiliations:** MRC Laboratory of Molecular Biology, Francis Crick Avenue, Cambridge CB2 0QH, UK

**Keywords:** Electron cryomicroscopy, Single-particle reconstruction, Gold, CryoEM, Protein structure, Magnification calibration, Pixel size

## Abstract

Determining the correct magnified pixel size of single-particle cryoEM micrographs is necessary to maximize resolution and enable accurate model building. Here we describe a simple and rapid procedure for determining the absolute magnification in an electron cryomicroscope to a precision of <0.5%. We show how to use the atomic lattice spacings of crystals of thin and readily available test specimens, such as gold, as an absolute reference to determine magnification for both room temperature and cryogenic imaging. We compare this method to other commonly used methods, and show that it provides comparable accuracy in spite of its simplicity. This magnification calibration method provides a definitive reference quantity for data analysis and processing, simplifies the combination of multiple datasets from different microscopes and detectors, and improves the accuracy with which the contrast transfer function of the microscope can be determined. We also provide an open source program, magCalEM, which can be used to accurately estimate the magnified pixel size of a cryoEM dataset ex post facto.

## Highlights

• Accurate magnification calibration is essential in cryoEM.
• We describe a procedure for using gold foils to calibrate the magnification of a TEM.
• The presence of both *fcc* and *hcp* gold lattices was discovered in commercial foils.
• An open source program for calibrating magnification, magCalEM, is provided.

## 1 Introduction and background

Magnification calibration is an important part of microscopy in general [1], [2], [3] and is a critical step in determining the structure of any biological specimen. Traditional optical microscopes have long been calibrated with gratings or rulers which are usually traceable by comparison to some absolute reference distance. In electron cryomicroscopy (cryoEM), often hundreds or thousands of images are collected at a fixed magnification in an automated way in order to provide enough data to determine a high resolution structure of a molecular specimen [4]. The magnification is not known a-priori for such a data collection session other than a nominal value as determined during the set-up and commissioning of the instrument.

In the modern era of digital electron detectors, the physical pixel size is fixed to a very high accuracy, which is usually set by the resolution of the manufacturing process used to make the sensor. The sensors used for electron detectors are typically manufactured using processes that can achieve a minimum feature size of 180–350 nm [5], [6], [7]. The error in pixel size over a 60 mm detector would thus be less than 1 part in $10^{5}$. Although the physical pixel size is fixed, it is the magnification via the projection lens system that varies in the modern electron microscope. When we refer to the region corresponding to one demagnified pixel on the specimen we will use the term “magnified pixel size”. So to be certain of the magnification in any particular micrograph, or across a dataset, one wishes to have an atomic scale “ruler” to determine the absolute magnification of the lens projection system, and thus the magnified pixel size in the digital image of the specimen.

**Fig. 1:**
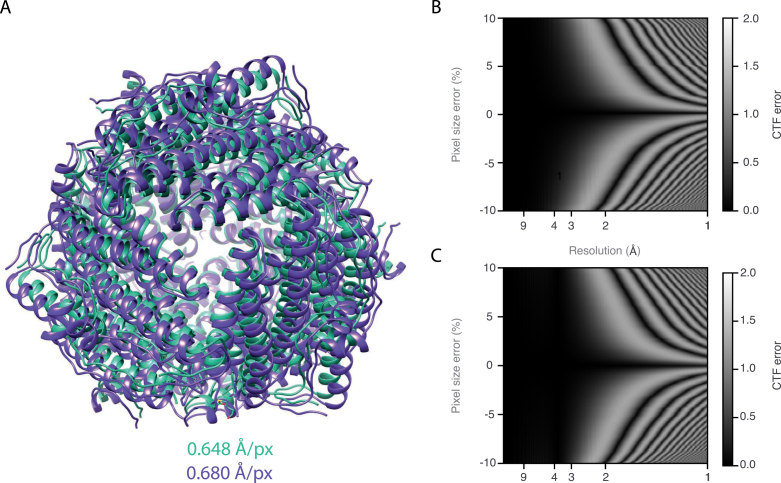
(A) shows two models of DPS, built using ModelAngelo [8] and refined using REFMAC5 [9], with the correct pixel size of 0.648 Å (green) and incorrect pixel size of 0.680 Å (purple). (B) and (C) show the error in the CTF as a function of spatial frequency and pixel size error for data collected at an accelerating voltage of 300 kV, a defocus of −1.5μm, and a $C_{s}$ of 2.7 mm. In (B), the defocus is kept at −1.5μm, whereas in (C) the defocus has been adjusted to minimize the error in the CTF.

It is essential that the magnification is determined accurately to allow an atomic model to be faithfully built (Fig. 1A), as model building programs are unable to correct for these errors. Since incorrect pixel sizes can in some cases erroneously improve model quality metrics such as clash scores [10], errors can be easily missed. Additionally, at the high resolutions now routinely achievable with single-particle cryoEM, even magnified pixel size errors of <1% cause substantial errors in fitting the phase of the contrast transfer function (CTF) (Fig. 1B), which in turn can reduce the resolution of the reconstructed map. The spatial frequency dependent phase shift of the electron wave caused by the objective lens is defined as

(1) $$\chi =\frac{\pi }{2}\left(2\Delta zq^{2}+C_{s}q^{4}\right)$$

where Δz is defocus (with underfocus as negative), q is the spatial frequency and $C_{s}$ is the spherical aberration coefficient. The contrast transfer function (CTF) is then

(2) $$\text{CTF}={\left(1-W^{2}\right)}^{\frac{1}{2}}\sin\chi -W\cos\chi$$

where W is the amplitude contrast term, which is approximately 0.04 for an accelerating voltage of 300 kV and without energy filtering [11]. The spatial frequency is related to the nominal magnified pixel size ($P_{nom}$) by the following equation

(3) $$q=\frac{r}{L\cdot P_{nom}}$$

where L is the full width of the Fourier transform in pixels, and r is the radius in pixels of the spatial frequency q in the Fourier transform. An error in magnified pixel size will thus result in incorrectly determined spatial frequencies and as a consequence, an inaccurate CTF (Fig. 1B). The error in magnified pixel size will be somewhat compensated since the fitted defocus will deviate from reality to counteract the error in q. However, since the $C_{s}$ and defocus terms are to different powers of q, this will only produce a good fit at low spatial frequencies (Fig. 1B). CTF errors at higher spatial frequencies will result in a loss of information extracted from the micrographs and thus a reduction in achievable resolution.

There are several methods commonly used by electron microscopists for magnification calibration, and these are outlined in the subsequent sections. These are: comparison to a known structure, $C_{s}$ refinement, the distance between lines on diffraction grating replicas, and the lattice spacing of crystalline materials.

### 1.1 Comparison to a known structure

One of the most common methods used in the context of cryoEM is to solve a standard protein structure to high resolution. The magnified pixel size can then be estimated by maximizing the cross correlation between this map and a map generated from a pre-existing high resolution X-ray structure, either manually using software such as Chimera [12] or automatically using EMDA [13].

### 1.2 $C_{s}$ refinement

Similarly, with the development of aberration correction during data processing [14], the magnified pixel size can be estimated from the symmetrical aberration coefficients. The aberration correction in RELION fits the $C_{s}$ such that it can be balanced with the defocus to minimize errors in the CTF. Using known values of $C_{s}$ and wavelength, these fits can be used to detect and estimate errors in magnification. The $Z_{4}^{0}$ coefficient, which is the 7th number in the “rlnEvenZernike” field of the RELION star file after aberration refinement, satisfies the equation:

(4) $$Z_{4}^{0}=6q^{4}-6q^{2}+1$$

We can ignore the lower order terms as they are cancelled by low-order Zernike coefficients. This means that the apparent $C_{s}$ is:

(5) $$C_{s\left(apparent\right)}=C_{s\left(true\right)}+\frac{Z_{4}^{0}}{\pi \lambda^{3}}$$

The correct magnified pixel size is then

(6) $$P_{true}=P_{nom}{\left(\frac{C_{s\left(true\right)}}{C_{s\left(apparent\right)}}\right)}^{\frac{1}{4}}$$

### 1.3 Diffraction grating replicas

Another common method is the use of diffraction grating replicas, which are shadow cast carbon replicas of parallel or crossed line gratings [15]. Since the real distance between these lines is known, the magnified distance between them in the image is used to measure the magnified pixel size. This method is fast and can be performed over a wide range of magnifications, but there are concerns that bending and distortions in the carbon foil can hamper its accuracy [16].

**Fig. 2:**
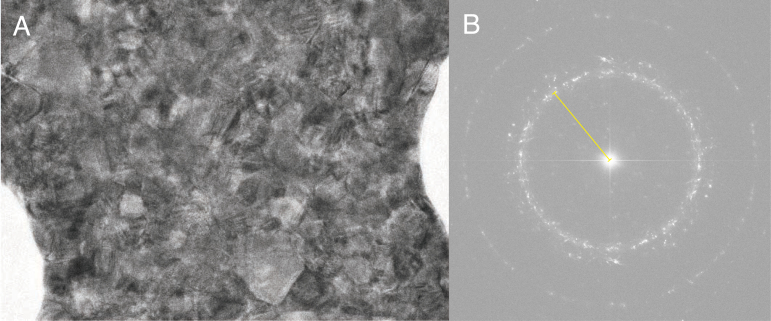
Demonstration of how to calculate the magnified pixel size from the Fourier transform of gold. In (A), a micrograph of polycrystalline gold foil from HexAuFoil grids gives a complete (111) ring in the Fourier transform shown in (B). The radius of the (111) ring can be used to estimate the magnified pixel size, given a gold lattice resolution of ≈2.35 Å.

### 1.4 Crystalline materials

Another suitable method is to use the lattice spacing of a crystalline material. Gold is an obvious choice for this since it is commonly used for the foil in cryoEM grids, including UltrAuFoil [17] and the more recent HexAuFoil [18], [19] grids. Gold is also one of the two materials recommended to be used for this purpose by the International Organization for Standardization [20]. The other recommended element is silver, although the lattice constants for silver are incorrectly listed as those for silicon in the table in Ref. [20]. The (111) reflection of pure gold diffracts to ≈2.35 Å resolution, meaning that the location of this peak in the Fourier transform of an image could be used to calibrate the magnified pixel size (Fig. 2). This necessitates that the magnified pixel size is less than ≈1.2 Å, although the peak can still be detected at lower magnifications on high efficiency detectors (up to ≈2 Å) as a result of aliasing. For lower magnifications, materials with larger lattice spacings can also be considered. Graphitized carbon has long been used as a calibration specimen for electron microscopy [21]. With its most visible lattice spacing being ≈3.4 Å resolution, graphitized carbon is a specimen well suited for the calibration of lower magnifications, but it is not as stable or robust as gold.

Here we investigate the accuracy of these methods for magnification calibration in electron cryomicroscopy. In particular, we determine several common pitfalls associated with using commercially available test specimens. We present a method for magnification calibration using a range of different commercially available and home-made test specimens. Additionally, we provide a simple program, magCalEM, allowing anyone to accurately measure their magnified pixel size after only a few minutes of processing time.

## 2 Materials and methods

### 2.1 Production of calibration specimens

The samples of *Mus musculus* heavy chain apoferritin were produced according to the procedure in Ref. [22]. After purification, 20 μL aliquots at a concentration of 10.8 mg/mL were flash froxen and stored at −80°C.

A K27 A mutant of DNA-binding protein from starved cells (DPS) from E. coli was purified as described elsewhere [23], [24], [25], [26] and stored at −80°C, at a concentration of 4.1 mg/mL in 20 μL aliquots. The sample was diluted to 1.0 mg/mL in 10 mM HEPES (pH 7.5), 100 mM NaCl, and 10 mM MgCl${}_{2}$ immediately before application to the grid.

Two different types of all-gold supports (UltrAuFoil R0.6/1.0, 300 mesh, Quantifoil, and HexAuFoil R0.3/0.3 made in-house) were exposed to a low energy plasma (Fischione 1070) comprising a mixture of argon and oxygen (9:1) for 90 s at 70% power to render them hydrophilic. The specimens were vitrified in a 4 °C cold room by plunge-freezing into a liquid-ethane cryostat [27] set to 93 K using a manual plunger of the Talmon type designed in-house [28]. A sample volume of 3 μL was pipetted onto the foil side of the grid and blotted for 10–15 s from the same side by Whatman No. 1 filter paper. The apoferritin sample was applied to the R0.6/1.0 300 mesh UltrAuFoil gold grids and the DPS to the R0.3/0.3 HexAuFoil gold grids. All samples were stored in liquid nitrogen until imaging in the electron microscope.

A simple procedure for preparing graphitized carbon calibration test specimens from readily available materials is given in Supplementary §1.

### 2.2 Data collection

Data were collected across several sessions on three different transmission electron microscopes (TEMs). Data on all biological specimens were collected on a TEM (FEI Titan Krios G3i electron cryomicroscope) operating at an accelerating voltage of 300 kV. The microscope was set to a nominal magnification of 130,000× and the camera was a K3 direct electron detector (Gatan) operating in counting mode. The flux was set to 44 e${}^{-}$/Å${}^{2}$/s, with an exposure time of 1.36 s to give a total fluence of 60 e${}^{-}$/Å${}^{2}$. Zero-loss energy filtering was performed using a Gatan Quantum energy-filter (GIF) with a slit width of 20 eV.

For calibration of the lattice constant of graphitized carbon grids, data were collected on a FEI Titan Krios G2 TEM at an accelerating voltage of 300 kV and a nominal magnification of 130,000×. The camera was a K3 direct electron detector (Gatan) operating in counting mode and zero-loss energy filtering was performed with a GIF and a slit width of 20 eV. The flux was 43 e${}^{-}$/Å${}^{2}$/s and the exposure time 8 s.

To calibrate the gold lattice constant at low temperatures, data were collected on a Tecnai Polara (FEI) using a Falcon 3 detector in integrating mode. The beam energy was 300 keV and nominal magnification 115,000×. The flux was 30 e${}^{-}$/Å${}^{2}$/s and the exposure time 8 s.

For measuring the variation in gold foil structure, samples were imaged on an FEI Titan Krios G2 TEM at an accelerating voltage of 300 kV and a nominal magnification of 96,000×. The camera was a Falcon 3 direct electron detector (Thermo Scientific) operating in linear mode and the total fluence was 84 e${}^{-}$/Å${}^{2}$. The temperature of the specimen was ≈80K. Diffraction patterns were collected on the same microscope, using a Ceta-M (Thermo Scientific) camera with a nominal camera length of 540 mm.

### 2.3 Data processing

To process the apoferritin dataset, 1224 tiff movies were imported into RELION 4.0 [29] with a pixel size of 0.646 Å and motion corrected with RELION’s own implementation of MotionCor2 [30]. CTF estimation was performed with CTFFIND-4.1.13 [31]. Particles were picked using RELION’s template-based algorithm and extracted into 400 × 400 pixel boxes. The templates were generated from 2D classes of manually picked particles. After an initial round of 2D classification, 253,849 particles were selected. Initial 3D auto-refinement gave a reconstruction with an estimated resolution of 1.9 Å. Three runs of CTF refinement [14] were performed next, with magnification anisotropy refined first, followed by optical aberrations, and finally per-particle defocus and per-micrograph astigmatism. The magnification anisotropy was measured as 1.7%. After 3D auto-refinement the resolution reached 1.6 Å. Bayesian polishing [32] was performed next to give a structure at 1.54 Å resolution. After a final round of CTF refinement and polishing, Ewald sphere correction [33], [34] was performed to give a final reconstruction at 1.52 Å resolution. To test the method of using CTF refinement for pixel size calibration, the dataset was reprocessed using different numbers of particles and an initial pixel size of 0.669 Å (Supplementary Table S1).

To process the DPS dataset, 4365 tiff movies were imported into RELION 4.0 with a pixel size of 0.646 Å. The same processing steps were performed as above, but particle picking was performed with crYOLO [35]. 412,902 particles were extracted into 256 × 256 pixel boxes, which became 234,690 particles after 2D classification. The resolution of the final reconstruction was 1.81 Å.

For the lattice spacing specimens, the movies were converted to MRC format [36] and motion corrected using Unblur [37].

## 3 Results

### 3.1 Lattice spacing calibration

To achieve results of a high accuracy, care must be taken when using lattice spacings to calibrate the magnification. There are several parameters that one must consider to ensure an accurate value is obtained. These parameters include: the presence of a mixed *fcc* and *hcp* gold lattice in the test foil, anisotropic magnification in the projection system, weak signal resulting in systematic underestimation of the magnified pixel size, and the temperature of the specimen.

**Fig. 3:**
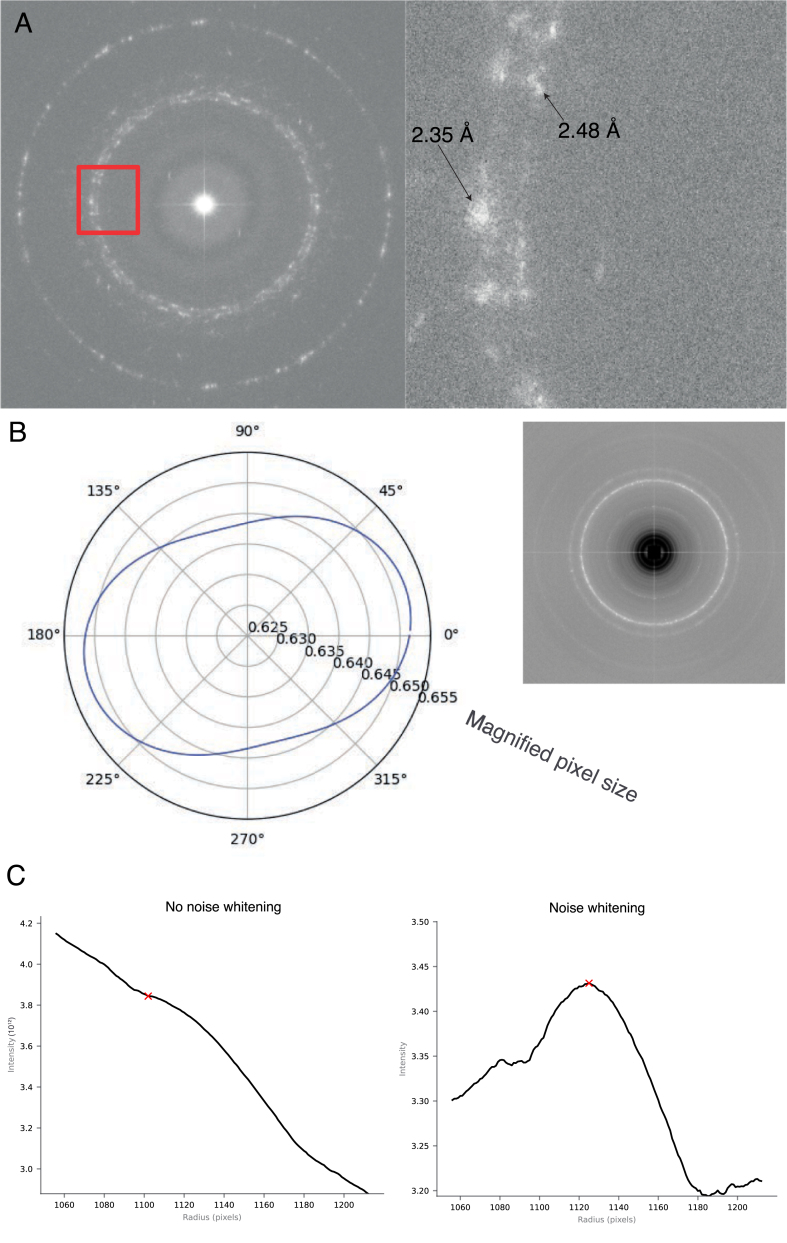
Issues in data processing of lattice spacing data that must be correctly handled. (A) The gold foil on UltrAuFoil grids displays two clear peaks at 2.35 Å and 2.48 Å. The inner peak at 2.48 Å is likely from *hcp* gold and selection of this results in a 5% error in magnified pixel size. (B) Although by eye the ring in the Fourier transform looks round, magCalEM measured significant anisotropic magnification. (C) Plots of the radial profile, centered on the true peak position. The peak of a weak signal is shifted towards the center when noise whitening is not employed (left). Noise whitening the power spectrum (right) prevents this.

#### 3.1.1 Mixed gold lattice

The gold foil on cryoEM grids is generally assumed to be pure *fcc* gold, with a lattice constant of ≈4.07 Å at room temperature. We noticed when using commercial UltrAuFoil grids that there were two distinct rings either side of 2.4 Å (at 2.35 and 2.48 Å, Fig. 3A). Choosing the incorrect peak would result in >5% error in the estimated magnified pixel size. This suggests that gold can have two forms, with the inner ring at 2.48 Å likely as a result of *hcp* gold (Section 3.3). It is therefore essential that multiple reflections are recorded so that the peak corresponding to pure *fcc* gold can be reliably selected. On the HexAuFoil grids, although some rare *hcp* gold reflections are observed, they are much weaker than the pure *fcc* gold reflections. It is likely that heating of UltrAuFoil grids during the manufacturing process, which is precluded in the manufacturing of HexAuFoil grids, is the reason for the presence of *hcp* gold.

#### 3.1.2 Magnification anisotropy

The magnified pixel size is not necessarily the same in all directions as a result of anisotropic magnification. This means that the spots in an FFT of an image of a polycrystalline lattice subtend an ellipse, not a circle (Fig. 3B). This can interfere with peak fitting and also necessitates a complete (111) ring; if a single reflection is used for calibration it is not known where on the ellipse it resides. In the example in (Fig. 3B), the estimated magnified pixel size could be 0.01 Å either side of the true value if only one reflection is selected for calibration.

#### 3.1.3 Weak signal

If the gold signal is not strong in the micrograph, finding the true reflection position becomes more challenging. The background intensity in the Fourier transform decreases with increasing frequency as a result of the detector MTF and low angle scattering. As a result, the peak position of the gold (111) reflection is shifted towards the centre of the Fourier transform. For a strong gold peak, this will make little difference, but data with weak reflections will be more severely affected. In the example in Fig. 3C, the peak of the pixel is shifted 20 pixels by noise whitening, resulting in a 0.01 Å reduction in the magnified pixel size estimate.

#### 3.1.4 Specimen temperature

The specimen temperature is important since it changes the lattice spacing of the material. For gold, the lattice constants at low temperatures have been previously published [38], [39]. Following these measurements, the lattice constant, a, in Ångstroms approximates a linear scaling according to

(7) $$a=4.0611\;\text{Å}+\left(5.67075\times 10^{-5}\;\text{Å}/\text{K}\right)T$$

where T is the temperature in Kelvin. However, the rate of decrease of a will decrease at lower temperatures, with a being approximately constant between 0 and 30K [40]. We measured the lattice constant of the gold foil on a HexAuFoil grid at a temperature of 13K, by first calibrating the magnification at 81K. We measured a lattice constant of 4.0636 Å, which is equivalent to a temperature of 43K in Eq. (7). As a result, the lattice constant is kept as 4.0636 Å for magnification calibration at temperatures below 43K.

For graphitized carbon, the lattice constant was only measured at 81K. On a different microscope session to that of the data in Table 1, the lattice constant for graphitized carbon was calibrated by fitting it to the same magnified pixel size given by the HexAuFoil grids. We measured the peak to be at 3.42±0.01 Å resolution, which corresponds to the interplanar distance in the graphite lattice. We measured a lattice constant of 5.92±0.01 Å for graphitized carbon at 81K, resulting in a (111) reflection at a resolution of 3.42 Å.

**Table 1:** Table of the measured magnified pixel sizes from one data collection session. The data processing procedures for the apoferritin and DPS datasets are described in Section 2.3. σ is the standard deviation and $\sigma_{\overset{¯}{x}}$ the standard error of the mean. The magnified pixel size for CTF refinement is chosen as the value from all of the particles. The lattice spacing method measurements were made with the program magCalEM, described in Supplementary §2. 100 micrographs were used for the HexAuFoil dataset and 10 micrographs for the UltrAuFoil and graphitized carbon.

Method	Grid type	Biological specimen	Estimated magnified pixel size (Å)	σ (Å)	$\sigma_{\overset{¯}{x}}$ (Å)
Lattice spacing	HexAuFoil gold	DPS	0.6484	0.0013	0.0001
Lattice spacing	UltrAuFoil gold	–	0.6473	0.0032	0.0011
Lattice spacing	Graphitized carbon	–	0.6471	0.0022	0.0007
Atomic model	HexAuFoil gold	DPS	0.6485	0.0015	0.0005
CTF refinement	UltrAuFoil gold	Apoferritin	0.6476	–	–
Line spacing	Cross grating grid	–	0.6587	–	–

### 3.2 Comparison between methods

To facilitate others to accurately use the lattice spacing method for magnification calibration, we have written a program called magCalEM, which is described in Supplementary §2. This program was used on data taken with the same imaging conditions on HexAuFoil, UltrAuFoil, and graphitized carbon grids, giving an magnified pixel size of 0.6484, 0.6473 and 0.6471 Å respectively (Table 1).

**Fig. 4:**
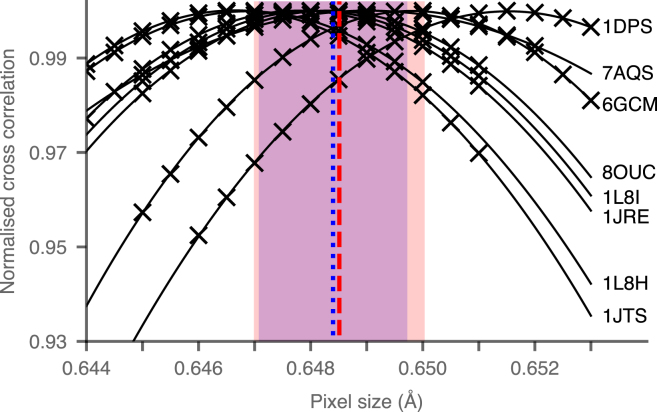
Cross correlation between maps generated from high resolution X-ray models of DPS and the cryoEM map. The cross correlation was measured as described in Section 2.3. The average is marked with the red vertical dashed line. The magnified pixel size estimated from gold HexAuFoil grids is marked by the blue dotted vertical line. The standard deviations are shown by the red and blue regions for the cross correlations and HexAuFoil measurements respectively.

For comparison, magnified pixel size was estimated using known protein structures by maximizing the cross correlation between a map generated from high resolution X-ray models of DPS (PDB: 1DPS, 1JRE, 1JTS, 1L8H, 1L8I, 6GCM, 7AQS, 8OUC) and the experimental cryoEM maps using Chimera [12]. The map was first generated from the model and then overlayed with the cryoEM map by eye. The ‘Fit in map’ function was then used to improve the alignment and measure the cross correlation. The pixel size of the cryoEM map was then adjusted and the ‘Fit in map’ used again to measure the cross correlation. The cross correlation values obtained are shown in Fig. 4. The average maximal cross correlation was at a pixel size of 0.6485 Å, with a standard deviation of 0.0015 Å.

Additionally, the apparent $C_{s}$ from CTF refinement was also used to estimate the magnified pixel size [41]. This CTF refinement method was applied to the apoferritin dataset processed with a deliberately incorrect starting pixel size of 0.669 Å, giving a magnified pixel size of 0.6476 Å. This was also performed for subsets of particles and hence maps of different resolutions. The results are in Supplementary Table S1, and have a mean of 0.6493 Å and standard deviation of 0.0039 Å.

When comparing the measured magnified pixel sizes from all methods, with the exception of the line spacing method at worse than 1.5% accuracy, the accuracy of all methods is similar, with all values within 0.5% of one another.

**Fig. 5:**
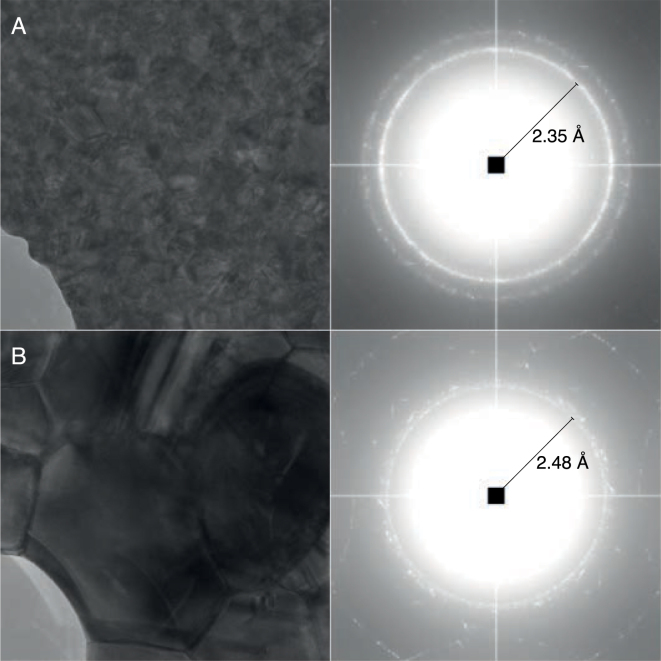
The effect of heating to 693 K on the gold foil on HexAuFoil gold grids. The summed Fourier transform from several foil regions on HexAuFoil grids (A) exhibits a strong peak at 2.35 Å resolution (111) and at 2.00 Å (200). After heat treatment (B), the grain size is noticeably larger and a peak at 2.48 Å is now the dominant peak in the Fourier transform.

### 3.3 Variation in the structure of commercial gold films

As described in Section 3.1, the images of gold foils from commercial UltrAuFoil grids had two rings in the Fourier transforms at ≈2.4 Å. The peak at 2.35 Å could be assigned to the (111) reflection of *fcc* gold, but the peak at 2.48 Å is inconsistent with a pure *fcc* gold lattice. It was thought that this second peak could be as a result of either: gold oxide, carbon dissolved in the gold, or as a result of *hcp* gold. To test the hypotheses, HexAuFoil and UltrAuFoil grids were sealed in glass vials of argon at a pressure of 2.2 Torr and heated on a hot plate to 693K for 5 min. This heating procedure in an anaerobic environment is expected to decompose the gold oxide [42]. In both cases, there was a clear increase in grain size of the gold, which is shown for the HexAuFoil grid (Fig. 5). Additionally, both types of grids had transformed such that the 2.48 Å resolution peak was now the dominant peak (Fig. 5B). Since any oxide should have been removed by heating under anaerobic conditions and the HexAuFoil grids have no carbon layer, it is likely that this peak is a result of *hcp* gold. The peak would then correspond to the (002) reflection of *hcp* gold, with lattice constants of a=2.88 Å and c=4.96 Å. These lattice constants are similar, albeit slightly higher, than previously published experimental results [43], [44].

**Fig. 6:**
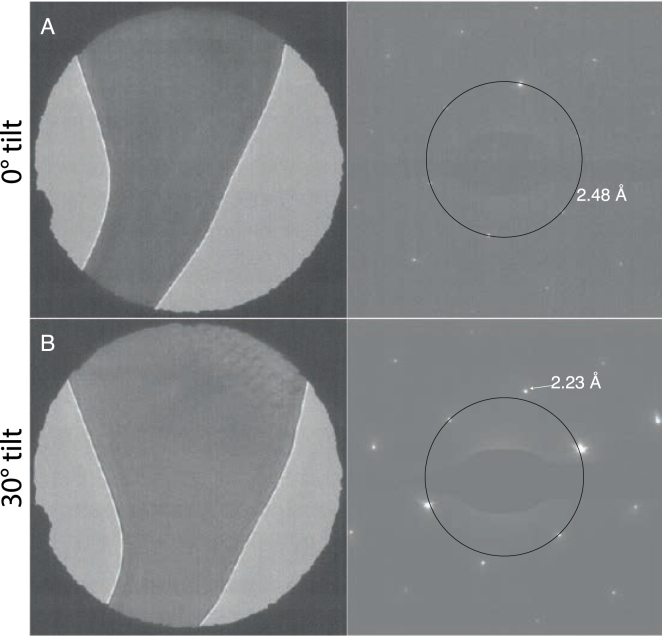
Selected area diffraction patterns from a large gold grain on the heat treated HexAuFoil grids with no tilt (A) and tilted to 30° (B). After tilting to 30°, a reflection at 2.23 Å resolution is now visible.

It is notable that the ($1\overset{¯}{1}\overset{¯}{1}$) reflection at 2.23 Å is absent in the Fourier transforms of the heated gold foil images. To investigate this, selected area electron diffraction patterns were collected of a *hcp* crystal on the heated HexAuFoil grids with no tilt and at 30° tilt (Fig. 6). With no tilt (Fig. 6A), the (002) reflection at 2.48 Å is clearly seen but there are no other reflections with a similar d spacing. After tilting to 30°, more nearby reflections, including the putative ($1\overset{¯}{1}\overset{¯}{1}$) reflection at 2.23 Å, can now also be seen. This suggests that the *hcp* [001] zone axis is oriented perpendicular to the foil, so the ($1\overset{¯}{1}\overset{¯}{1}$) reflections are not visible until the crystal is tilted.

## 4 Discussion

Given that the measured magnified pixel sizes from the gold foils, the high resolution protein structures, and the graphitized carbon are all within <0.5%, it is likely that all of these methods are suitable for accurate magnification calibration in cryoEM. The line spacing method gave an error of >1%, and is thus unsuitable for accurate magnification calibration. We can only speculate as to why the line spacing method was not accurate, but bending of the carbon foil or errors in fitting the center of the lines are possible explanations.

Using a high resolution protein structure does produce accurate results, but several X-ray models must be used to produce results of equal accuracy to those achieved using the lattice constant measured from the HexAuFoil grids. These X-rays models will not exist for the majority of targets, and cryoEM models are unsuitable because they may have the incorrect magnified pixel size. When also including the time to set-up as well as collect data, this method is considerably more time consuming than the others. Furthermore, this method relies on the magnification not changing between the time of calibration and data collection, which could be many months or even years in a busy facility. A much faster calibration method is required to allow routine magnification calibration.

The output from CTF refinement proved to be accurate to <0.5% for these datasets. This does, however, not only require a high-resolution structure to be determined, but the $C_{s}$ refinement must have been successful, which is less likely as the resolution decreases (Supplementary Table S1). Additionally, one must be mindful that this method will not provide accurate results if the magnified pixel size was rescaled during data processing or if the energy of the electron beam was incorrect.

A perhaps obvious choice for calibration specimen would be to use the gold on a cross grating grid. Unfortunately, the commercially available cross grating grids are shadowed with not only gold, but also palladium, which has a smaller lattice constant and its (111) peak is thus at a higher resolution. Attempts were made to use the diffraction from the cross grating grid to calibrate the magnification, but it consistently gave a result 1%–2% too high. This is likely because the presence of palladium, in an ≈60:40 ratio of gold to palladium [15], shifts the peak to a higher resolution, as was described in [22].

Pure gold foils have an advantage over other specimens in that they can be a part of the same specimen support that is being used to collect data, so no extra preparation is required. Another advantage is that if the edge of the hole is visible in the images, which is likely if small hole HexAufoil grids are being used [18], [45] or if data are collected off-center and/or with multiple shots per hole, then no extra images are necessary. By using data collected alongside single-particle images, it ensures that the lens currents and alignments are identical to that of the data. The magnified pixel size can simply be estimated after or even during a standard data collection. This is particularly useful if the magnified pixel size is not always the same at a given nominal magnification, which could be the case for several reasons including the following: a. If the illuminating beam is not parallel, images taken at different defocus values will have different magnifications, and the amount of change with defocus will increase with increasing amounts of convergence/divergence. The magnification change due to non-parallel illumination can sometimes be corrected in software for a particular data set by separating the micrographs according to defocus value and treating them separately during processing (e.g. using optical groups in RELION), but one will always be better off starting this process with a well calibrated value for the magnification at the average defocus value used for data collection. b. Errors in the tuning of the energy filter can result in magnification errors. c. By comparing values from 4 Krios microscopes of increasing age at the LMB, we have noticed that the degree of asymmetric magnification usually increases over the lifespan of a microscope, possibly due to the build-up of contamination in the projection system.

When calibrating magnification using images of the gold foil from HexAuFoil grids and UltrAuFoil grids, the data from the HexAuFoil grids were easier to process than those from the UltrAuFoil grids. This was because the crystal size in the former is around 10 times smaller [18] so a complete (111) ring is obtained from a smaller image area. The issue with multiple gold lattice types that was present in UltrAuFoil grids was also not seen in any HexAuFoil grids.

A magnified pixel size of 0.648 Å was used in this study for comparison between methods, but we have demonstrated that a pixel size of 1.33 Å can be calibrated on a HexAuFoil gold grid (Figure S4), and pixel sizes as high as ≈2.0 Å should be possible to calibrate with this method. Since the graphitized carbon grids were also shown to produce reasonably accurate results, they are a viable option for magnified pixel sizes between 2.0–3.0 Å.

We recommend that >10 micrographs of the foil from a dry HexAuFoil grid are taken at each magnification by facility staff for initial calibration. This should be repeated after any major changes to the optics or detector. It is also useful to occasionally (on the timescale of months) check that the magnification has not changed as this can be a sign of other problems with the optics. For users of gold foil specimen supports, magCalEM enables them to check the magnification of an already collected dataset to ensure it is correct and models can be built accurately.

## 5 Conclusions

Magnification calibration in cryoEM often requires more effort than it should. Accurately calibrating the magnified pixel size for each dataset results in easier and more accurate data processing as well as simplifying the processing of multiple datasets when combined. Here we have presented a simple method for accurate atomic-resolution magnification calibration in cryoEM using polycrystalline gold as an absolute reference standard. We have demonstrated that this method can estimate the magnified pixel size to an accuracy of <0.5% in only a matter of minutes of processing time. We have provided a program with both command line and graphical user interfaces, enabling others to easily do the same.

## Declaration of competing interest

The authors declare that they have no known competing financial interests or personal relationships that could have appeared to influence the work reported in this paper.

## Data Availability

The final reconstructed maps for DPS (EMD-17992) and apoferritin (EMD-17995) are deposited in the Electron Microscopy Data Bank. The code is available at www.mrc-lmb.cam.ac.uk/crusso/ and also available to install at www.pypi.org/project/magCalEM.

## Appendix A Supplementary data

MMC S1 Protocol to make graphitized carbon grids. Details of magCalEM. X-ray diffraction. Aliasing example. CTFRefine results.
