# Supplementary material for: Accurate magnification determination for cryoEM using gold
Source: Ultramicroscopy. 2024 Feb;256:None. doi: 10.1016/j.ultramic.2023.113883 (PMC10782223; doi:10.1016/j.ultramic.2023.113883)
Supplement: MMC S1 — Protocol to make graphitized carbon grids. Details of magCalEM. X-ray diffraction. Aliasing example. CTFRefine results. [file mmc1.pdf]

Supplementary information

1. Preparing graphitized carbon specimen

The following is a procedure for making graphitized carbon grids for use in microscope alignment and magnification calibration. These grids can be used to calibrate lower magnifications with a nominal pixel size smaller than $\approx$3.0 Å. Their strong Thon ring pattern also provides a particularly useful tool for the correction of objective astigmatism.

1.1. Materials

- Graphitized Carbon Black (GCB) - e.g. Supelco Small Particle GCB Adsorbant
- Chloroform (high purity preferred)
- Holey TEM Grids - e.g. Quantifoil Cu 300 mesh
- Filter Paper
- Tip Sonicator
- Scales - Accurate to +/- 1 mg
- Glassware - test tubes/pipettes
- High precision tweezers - e.g Dumont N5

1.2. Procedure

1. Weigh out GCB powder and add to an equal weight of chloroform to form a 1 mg/mL solution, shake gently to mix.
2. Sonicate solution using a tip sonicator for around 30 seconds at 25 W to form an even suspension of particles.

3. Dilute solution a further 20 times with chloroform to form a 0.05 mg/mL solution.
4. Shake gently to mix, then sonicate again briefly.
5. Pipette 3 µl of the 0.05 mg/mL solution onto each TEM grid.
6. Leave solution to settle on grid for a few seconds before blotting away excess solution from below with filter paper.
7. Allow each grid to dry for around 30 seconds before storing. Grids can be stored for long periods in a standard room temperature environment.

1.3. Common pitfalls

- Use glassware where possible; avoid plastic as this can be dissolved by the chloroform leaving visible polymer contamination on grids.

- Avoid the use of polar solvents for making GCB suspensions. Acetone, ethanol or even water can de-graphitize the GCB (particularly when energy is added to solution via sonication). These carbon particles will then result in beam induced contamination during electron irradiation.

1.4. Expected Results and Usage

Grids should show an even dispersion of well separated particles, as shown in Figure S1A. To perform microscope calibration locate a suitably large crystal spanning a grid hole and increase magnification until internal "onion" structure of the particles can be observed (Figure S1B).

2. Program description

A simple program to estimate magnified pixel size was written in Python and can be used using a command line interface or a simple graphical user interface (Figure S2). It is available on www.mrc-lmb.cam.ac.uk/crusso/ and available to install at www.pypi.org/project/magCalEM. The organisation of the code into separate scripts should also allow straightforward integration into existing processing pipelines. The workflow for estimating the magnified pixel size is shown in Figure S3. Firstly, the user will import their data (single frame

micrographs) and define important parameters such as the temperature, the material, and a rough estimate of the magnified pixel size. Power spectra are then calculated from these images. If the estimated magnified pixel size is more than half the desired reflection's resolution, the peak will be beyond Nyquist frequency. In this case, aliasing can be exploited and the edges of the power spectra are mirrored to produce a complete ring in the Fourier transform (Figure S4).

The power spectra can then be noise whitened by either dividing by the detector modulation transfer function, or by using images containing none of the material being used for calibration (i.e. either blank images or images of amorphous ice). To do this, power spectra are produced from the blank/ice images and the radial profiles are calculated. The radial profiles are then smoothed using the Savitzky-Golay filter [1] and averaged to reduce noise. Next, power spectra of the data are divided by the average radial profile of the noise power. After noise whitening, batches of images are combined to ensure that there is a complete ring of the desired reflection in all noise whitened power spectra.

After producing noise whitened power spectra, the magnification anisotropy is measured. At least two methods have been proposed on how to use diffraction from polycsrystalline specimens to do this [2, 3], and we use a method similar to the former. Using a sum of all noise whitened power spectra, we calculate the magnified pixel sizes of 2 degree wedges, totalling 90 pixel size estimates in the 180 degree range of the power spectrum. Data points are plotted at each pixel size and an ellipse is fitted to these, with the parameters of the ellipse adjusted using least squares fitting (Figure 3B). If magnification anisotropy is present, the elliptical parameters are then used to stretch the noise whitened power spectra along the minor axis using bilinear interpolation.

Next, radial profiles are calculated from each of the power spectra and these are again smoothed with the Savitzky-Golay filter to reduce the impact of noise. The peak of the radial profile is fitted to a Voigt profile, and this radius is then converted to a magnified pixel size. The programs outputs the measured magnified pixel size and the estimated error. Additionally, the average power

spectrum is displayed with the measured magnified pixel size plotted, allowing the user to visually inspect that the calibration was successful.

3. X-ray diffraction on gold

X-ray diffraction was used to verify the thermal contraction of gold from room to cryogenic temperature. A length of gold wire (0.2 mm, 99.99% pure, Electron Microscopy Sciences) was glued as received onto X-ray sample mount. The wire was aligned with the 2θ centre before beginning data collection and remained unmoved or rotated during and between exposures. A beam of CuK_α radiation of wavelength 1.5418 Å was diffracted by the gold onto a Mar345 detector at a distance of 150 mm where both the (111) and (200) gold lattice reflections could be resolved. The detector was then moved away from the sample in steps of 5 mm taking 120 second exposures until first the (200) and then the (111) rings could no longer be detected. Room temperature data was collected in this manner at 298 ± 3K before cooling the sample to 100 ± 3K using Oxford Cryosystems cryostream 600 and collecting a second dataset using the same method. 2D diffraction patterns were converted to 1D diffraction plots using Fit2d software [4]. Diffraction peaks were fitted using Python3 lmfit least squares fitting. Sample displacement error was modelled and 2θ peak positions corrected accordingly [5].

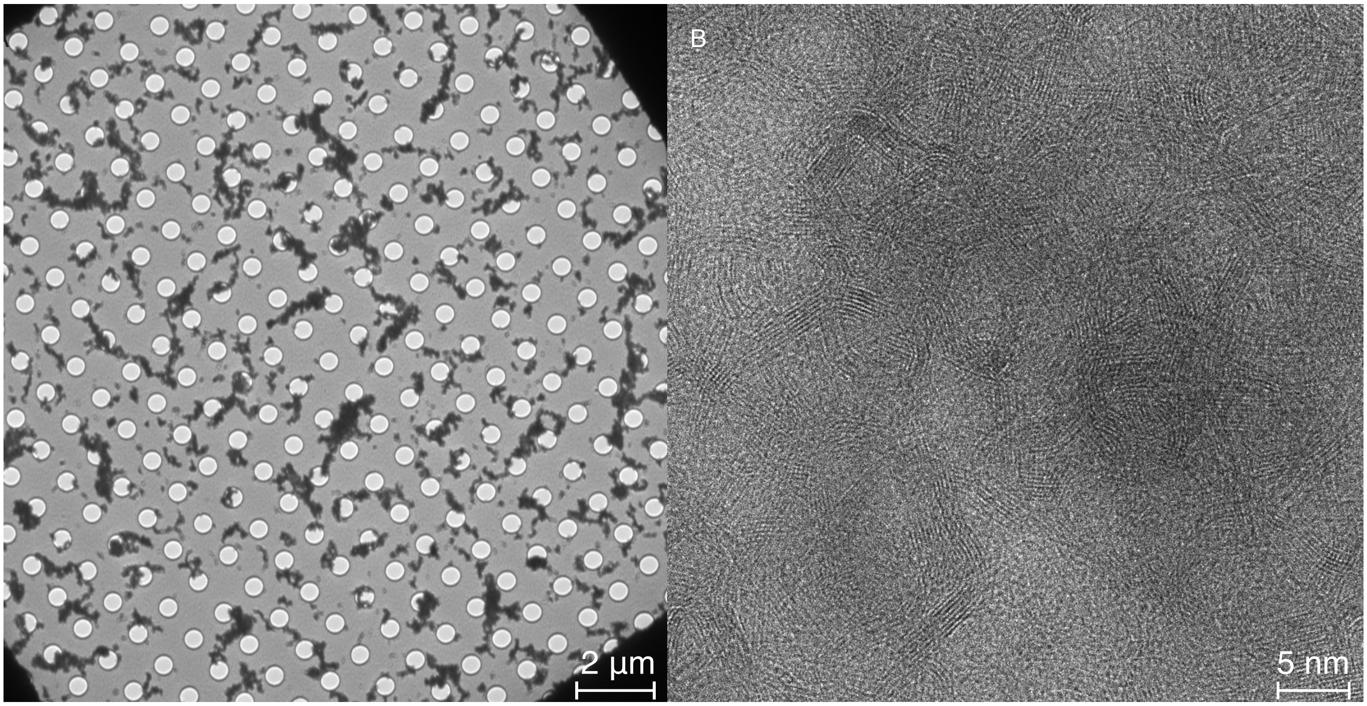

Figure S1: Low magnification (A) and high magnification (B) TEM images of a graphitized carbon grid.

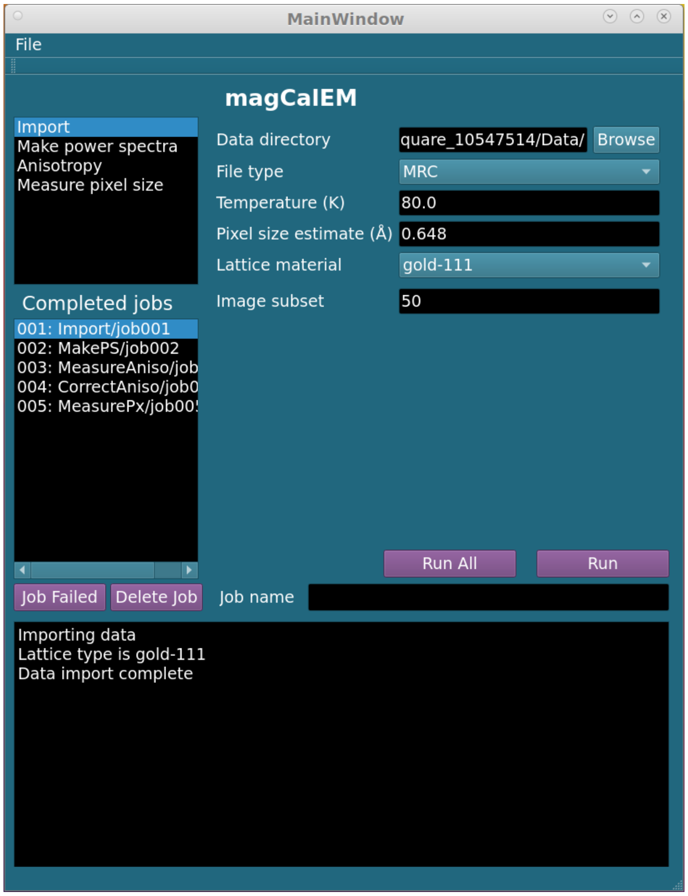

Figure S2: The main window of the magCalEM graphical user interface. All jobs can be run from this window and the output of previous jobs can also be selected and displayed.

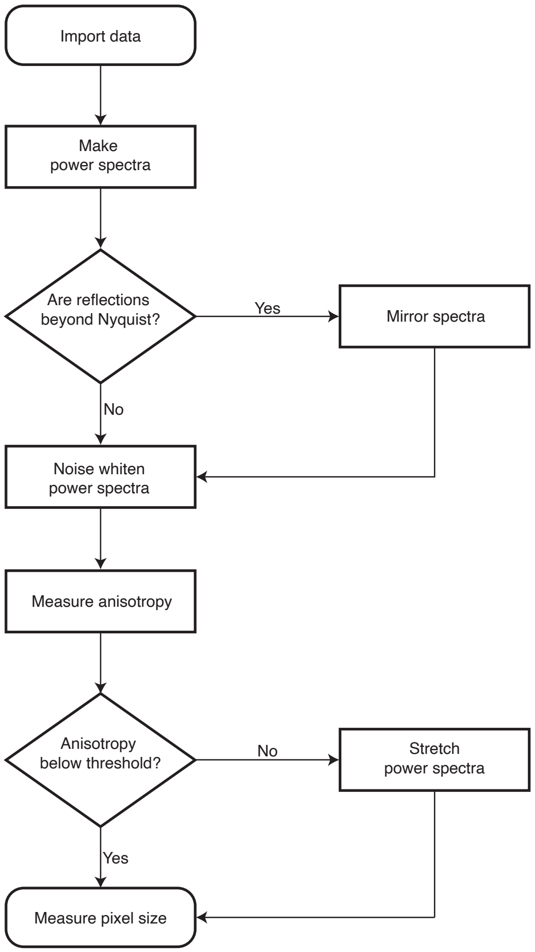

Figure S3: Workflow of the program to measure magnified pixel size from the lattice spacing of a material. The data is first imported and power spectra are calculated for each image. If the estimated magnified pixel size is beyond Nyquist frequency, the spectra is mirrored to produce a ring from the aliased signal. Magnification anisotropy is measured and corrected for if it is above 0.5%. Finally, the magnified pixel size is measured from the average peak position in the power spectra.

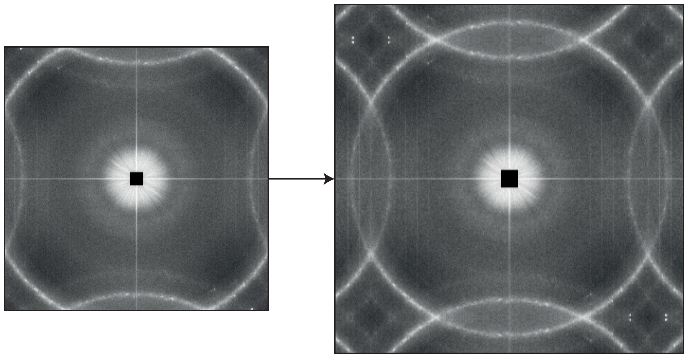

Figure S4: Example of how aliasing can be utilised to calibrate the magnification when the reflections are beyond Nyquist frequency. The sample is a HexAuFoil grid and the magnified pixel size was measured as 1.33 Å.

Number of particles	Resolution (Å)	Estimated magnified pixel size (Å)
159	3.92	0.6493
163	3.55	0.6497
279	2.95	0.6487
398	2.95	0.6497
428	3.39	0.6374
540	2.81	0.6489
1962	2.47	0.6529
2423	2.39	0.6509
4092	2.19	0.6521
4164	2.14	0.6524
8669	2.07	0.6512
10032	2.01	0.6505
212623	1.79	0.6476

Table S1: The estimated magnified pixel size from CtfRefine of an apoferritin dataset split into different numbers of particles. The data processing procedure is described in §2.3 of the main text.

References

[1] A. Savitzky, M. J. E. Golay, Smoothing and differentiation of data by simplified least squares procedures., Analytical Chemistry 36 (1964) 1627–1639.

[2] J. Zhao, M. A. Brubaker, S. Benlekbir, J. L. Rubinstein, Description and comparison of algorithms for correcting anisotropic magnification in cryo-EM images, Journal of Structural Biology 192 (2015) 209–215.

[3] T. Grant, N. Grigorieff, Automatic estimation and correction of anisotropic magnification distortion in electron microscopes, Journal of Structural Biology 192 (2015) 204–208.

[4] A. Hammersley, et al., FIT2D: an introduction and overview, European Synchrotron Radiation Facility Internal Report ESRF97HA02T 68 (1997) 58.

[5] L. McCusker, R. Von Dreele, D. Cox, D. Louër, P. Scardi, Rietveld refinement guidelines, Journal of Applied Crystallography 32 (1) (1999) 36–50.
